# Molecular Phenotypes of Unifocal, Multifocal, and Diffuse Invasive Breast Carcinomas

**DOI:** 10.4061/2011/480960

**Published:** 2010-11-03

**Authors:** Tibor Tot, Gyula Pekár, Syster Hofmeyer, Maria Gere, Miklós Tarján, Dan Hellberg, David Lindquist

**Affiliations:** ^1^Department of Pathology and Clinical Cytology, Central Hospital Falun, S-791 82 Falun, Sweden; ^2^Center for Clinical Research Dalarna, Uppsala University, Nissers Väg 3, 79181 Falun, Sweden

## Abstract

We analyzed the subgross distribution of the invasive component in 875 consecutive cases of breast carcinomas using large-format histology sections and compared the immunophenotype (estrogen and progesterone receptor expression, HER2 overexpression and expression of basal-like markers, CK5/6, CK14, and epidermal growth factor receptor) in unifocal, multifocal, and diffuse tumors. Histology grade and lymph node status were also analyzed. Unifocal invasive carcinomas comprised 58.6% (513/875), multifocal invasive carcinomas 36.5% (319/875), and diffuse invasive carcinomas 4.9% (43/875) of the cases. The proportion of lymph node-positive cases was significantly higher in multifocal and diffuse carcinomas compared to unifocal cancers, but no other statistically significant differences could be verified between these tumor categories. Histological multifocality and diffuse distribution of the invasive tumor component seem to be negative morphologic prognostic parameters in breast carcinomas, independent of the molecular phenotype.

## 1. Introduction

Breast carcinoma is a heterogeneous group of diseases; individual cases deviate from each other in morphology, phenotype, and prognosis. Using DNA microarray technique and cluster analysis, five distinct genetic types of the disease were delineated: luminal A, luminal B, HER2 positive, basal-like, and normal breast like tumors [1, 2]. These tumor subtypes can also be identified with sufficient accuracy during routine diagnosis, using a simple panel of immunohistochemical markers, including antibodies tracing estrogen receptors (ER), progesterone receptors (PR), c-erbB-2 oncoprotein overexpression (HER2), and some myoepithelial markers [3, 4]. The recommended myoepithelial markers for delineating the basal-like tumors vary in different studies, cytokeratin (CK)5/6, CK14, CK17, and/or epidermal growth factor receptor (EGFR) being used most often [3, 5–8]. Significant differences in survival of patients with different molecular subtypes of breast carcinoma have been recently evidenced; Luminal A tumors have a significantly better 5- and 10-year survival compared to luminal B, HER2 positive, basal-like, and unclassified tumors [9].

Using large-format histologic sections in diagnostic routine, we have repeatedly evidenced that breast carcinoma has a complex subgross morphology with a considerable proportion of the tumors being either multifocal or diffuse [10–13]. The most recent studies on breast cancer multifocality indicate that multifocality and diffuse distribution of the invasive tumor component represent survival-related negative prognostic parameters [14–17]. As we have not found corresponding data in the literature, we designed the present study with the aim to analyze the relation of subgross appearance of the lesions (unifocal, multifocal, or diffuse distribution of the invasive component) and some phenotypic tumor features, such as ER, PR, and HER2 expression, basal-like phenotype, and histology grade. We focused on the invasive component of the tumor and did not analyze the distribution of the in situ ductal or lobular components in this study. We also tested the relation of the subgross histologic distribution of the lesions to presence of lymph node metastasis (LNM).

## 2. Materials and Methods

### 2.1. Study Population

This study is a retrospective analysis of 875 consecutive breast carcinoma cases diagnosed at the Department of Pathology and Clinical Cytology of the Central Hospital in Falun, Sweden, during the period January 2005−December 2009. Patients with recurrent breast carcinomas that were diagnosed before the study period were excluded. We also excluded purely in situ carcinomas (132 cases), microinvasive (<1 mm) carcinomas, and carcinomas which were not routinely stained for the immunohistochemical markers listed below. The subgross parameters, histology grade, LNM, ER, PR, and HER2 status were analyzed during the entire study period. The basal-like phenotype was routinely assessed from September 2006 to the end of the study period. The study was approved by the Regional Ethical Committee in Uppsala-Örebro region.

### 2.2. Large-Section Histopathology

All specimens were prepared by the method of large-section histopathology, which has been a routine procedure in our laboratory since 1982. The method has been described in detail elsewhere [18]. Briefly, all cases were discussed by a preoperative tumor board, and the radiological (mammography, ultrasound, and magnetic resonance imaging) appearance was registered, including the extent and distribution. This information, together with the whole-specimen radiograph received with the surgical specimen, guided the pathologist during the workup. The sector-resection specimens were sliced into 3-4 mm-thick tissue slices parallel to the pectoralis fascia. The slices were also radiographed. One to five of the most representative slices (measuring up to 9 × 8 cm) were selected for embedding into large paraffin blocks. Larger slices were bisected and embedded into separate blocks. Mastectomy specimens were sliced perpendicular to the pectoralis fascia to visualize the surgical margin in one histological plane. All cases were discussed again on postoperative tumor board to check the concordance of the radiological and histological findings. Most cases which were discrepant in favour of radiology findings were solved with additional sampling of the specimen for histological analysis.

### 2.3. Immunohistochemistry

The largest invasive tumor focus was sampled for routine immunostaining. The following antibodies were used: ER (Ventana Medical Systems, clone : SP1, 1 : 200), PR (Dako, clone : PgR 636, 1 : 50), CK5/6 (Dako, clone : D5/16 B4, 1 : 100), CK14 (Novocastra, clone : LL002, 1 : 20), EGFR (Dako, clone : E 30, 1 : 25), and HER2 (Dako, code A 0485, 1 : 250). Additional foci were only stained in selected cases. Nuclear staining >10% of the tumor cells were the criterion of ER and PR positivity, cytoplasmic staining in >10% for CK5/6 and CK14 positivity, and membranous and cytoplasmic staining in >10% of the tumor cells for EGFR positivity. HER2 positivity was assessed in accordance with the criteria of the manufacturer; all 2+ equivocal cases underwent fluorescence in situ hybridisation test.

### 2.4. Diagnostic Criteria

**Figure 1:**
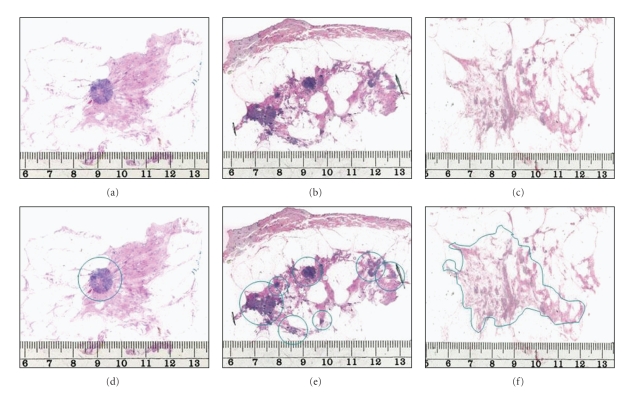
Typical cases of unifocal (left), multifocal (central), and diffuse (right) invasive breast carcinomas documented in large-format histology sections. The lesions are marked in the lower images.

The distributions of the invasive component and of the in situ component of the same lesion were determined separately. For the purpose of the present study, the tumors were classified based on the distribution of the invasive lesions. They were considered to be “unifocal” if only one invasive focus could be observed in the large sections, with the tumor focus containing or not containing an in situ component. “Multifocal” invasive lesions were characterized by the presence of multiple well-delineated invasive tumor foci separated from each other by uninvolved breast tissue, regardless of the distance between the foci. Tumors that were dispersed over a large area in the section, much like a spider's web, with no distinct tumor mass were classified as “diffuse.” The size of the diffuse tumors was equal to the extent of the disease in many cases and was rather comparable to the extent of the disease in multifocal cases than to the size of the individual foci. When the distribution of the lesions was assessed, in each case, an attempt was made to summarize the findings in different levels of the large sections to reconstruct the in vivo situation before operation. Detailed correlation between radiological and pathological findings was essential. If a secondary surgical intervention was performed in addition to the primary sector resection, an attempt was made to summarize the findings in the entire excised tissue. However, sector resection specimens (average size of 9 × 6 cm) were sufficient for categorizing the findings in most cases. Typical cases of unifocal, multifocal, and diffuse invasive breast carcinomas are illustrated in Figure 1.

LNM was defined as presence of metastatic deposit(s) in at least one of the lymph nodes of the case, irrespective on the size of the deposit(s). Both sentinel and nonsentinel nodes were assessed on routinely stained sections. The sentinel lymph nodes were additionally stained on CK8/18 (BD Biosciences, clone Cam 5.2, 1 : 50). Tumors expressing at least one of the basal (myoepithelial) markers (CK5/6, CK14, EGFR) in at least one of the invasive tumor foci were categorized as basal-like tumors. Triple-negative tumors were defined as negative for all of the following markers: ER, PR, and HER2. The tumors were graded according to the Notthingham (Bloom-Richardson-Elston-Ellis) grading system [19]. Tumor size was defined as the largest dimension of the largest invasive focus.

### 2.5. Study Execution

All of the large histological sections belonging to this series (average number of sections per case 6, range of 1–34) were reviewed for the purposes of postoperative tumor board. Histological data, including the distribution of lesions, were determined according to the diagnostic criteria described above and registered in a database. “Multicentricity,” which is defined as the presence of malignant structures in different quadrants of the same breast, was not analyzed because it represents a clinical and/or radiological parameter. Phenotypic parameters were obtained from the department's database. The statistical analysis (comparison of proportion using chi-square test) was carried out using commercially available software (MedCalc statistics for biomedical research, MedCalc Software, Belgium), with *P*-values <.01 regarded significant.

## 3. Results

The distribution of the invasive lesions could be accurately analyzed in 875 invasive cases. Unifocal invasive carcinomas comprised 58.6% (513/875), multifocal invasive carcinomas 36.5% (319/875), and diffuse invasive carcinomas 4.9% (43/875) of the cases. 

Histology grade was determined in 870 of the invasive carcinomas of which 22.8% (198/870) were grade 3. As demonstrated in Table 1, there were 115 unifocal, 80 multifocal, and 3 diffuse grade 3 invasive carcinomas. The percentage of unifocal and multifocal grade 3 cases were very similar (22.6% versus 25.2%). The proportion of grade 3 cases among diffuse invasive carcinomas was only 7.0%, but the difference was not statistically significant as only 3 such cases were found. 

Tumor size could be accurately assessed in 511 unifocal (average size 16.0 mm, range 3–70 mm), 315 multifocal (average size 19.5 mm, range 2–60 mm), and in 41 diffuse (average size 45.6 mm, range 20–85 mm) invasive carcinomas. This difference was not statistically significant when unifocal and multifocal tumors were compared (*P* = .7375), but the differences became significant when the diffuse group was added to analysis (*P* = .0003). 

ER status was assessed in 865 invasive breast carcinomas. There were 83.4% (721/865) ER-positive and 16.6% (144/865) ER-negative cases. There were 83.1% (423/509) unifocal, 81.8% (256/313) multifocal, and 97.7% (42/43) diffuse ER-positive invasive cancers. The differences were statistically not significant. 

PR was assessed in 855 cases. There were 65.1% (557/855) PR-positive and 34.9% (298/855) PR-negative cases. 66.3% (331/499) of the unifocal, 64.9% (203/313) of the multifocal, and 53.5% (23/43) of the diffuse invasive carcinomas were PR positive. The differences were statistically not significant.

During the study period, 858 invasive breast carcinomas were tested for HER2 overexpression and 11.4% (98/858) were found to be HER2 positive: 9.6% (48/501) unifocal, 15.2% (48/315) multifocal, and 4.8% (2/42) diffuse tumors. The differences were statistically not significant.

The proportion of triple negative cases was 10.5% (90/854) in the present series, 11.2% (56/499) among the unifocal, 10.5% (33/313) among the multifocal, and 2.4% (1/42) among the diffuse cases. The differences were statistically not significant. 

Carcinomas were routinely stained for basal markers in 518 cases. Of those, 11.6% (60/518) expressed basal-like phenotype, 12.5% (37/296) of the unifocal, 11.3% (22/195) of the multifocal, and 3.7% (1/27) of the diffuse tumors. The differences were statistically not significant.

LNM was determined in all cases of the present series of invasive breast carcinomas and 38.5% (337/875) of the cases had some form of metastatic tumor spread (including macrometastases, micrometastases, and isolated cancer cells/cellgroups). The proportion of lymph node positive cases was 27.3% (140/513) in the group of unifocal cancers, 54.2% (173/319) in the group of multifocal cancers, and 55.8% (24/43) in the group of diffuse tumors. These differences were statistically highly significant.

## 4. Discussion

Breast cancer is a disease with wide variation in subgross morphology. Tumor multifocality has been evidenced in a substantial proportion of the cases in early whole organ studies and is seen in >30% in the series of cases documented with large-format histology slides [10–13]. In addition, 5% of the invasive carcinomas exhibit a diffuse, spider's web-like growth pattern [14]. Modern radiology methods, especially if used in combination (multimodality approach), are able to indicate the growth pattern of the tumor in the vast majority of the cases [18]. Detailed radiological-pathological correlation and regular use of large-format histology slides enables the breast pathologist to correctly assess tumor size, disease extent, and multifocality or diffuse growth and to confirm or correct the preoperative radiological results. 

The prognostic significance of tumor multifocality has recently received special attention as, in contrast to some previous publications [20], recent long-term followup studies have demonstrated significantly lower breast cancer-specific survival in multifocal than in unifocal tumors [15–17]. Multifocality seems to be a negative morphologic parameter independent of treatment modalities [16]. Diffuse invasive carcinomas have an even worse prognosis than the multifocal tumors [14, 17]. The question arises whether differences in survival between unifocal, multifocal, and diffuse breast carcinomas can be explained with differences in their molecular phenotype. The present study was carried out on a recent series of cases, thus survival of the patients could not be tested. 

The greater metastatic capacity of multifocal and diffuse tumors compared to the unifocal ones has been repeatedly proven in independent studies [10–12, 20–22] and was also confirmed in the present study. In fact, this was the only statistically significant difference between unifocal versus multifocal and unifocal versus diffuse invasive carcinomas in the present series.

The relation of tumor multifocality and tumor phenotype is rarely studied in the literature. Histology grade, ER, PR, and HER2 status represent well-established, routinely assessed morphological prognostic parameters [23, 24]. According to our results, no significant differences could be demonstrated between these tumor categories with respect to histology grade, ER, PR, and HER2 status. The same finding has been reported by Litton et al., but their study was limited to women ≤35 years [25]. In the study of Oh et al., no significant differences were found between unifocal and multifocal/multicentric tumors regarding nuclear grade and ER status [26]. 

While multifocal breast carcinomas had a tendency to show more unfavourable phenotype, although statistically not significantly different, compared to the unifocal tumors, the diffuse invasive carcinomas exhibited an opposite tendency. They were less often ER negative, less often triple negative, less often HER2 positive, or basal-like than the unifocal cancers. Although this is a remarkable phenomenon as these tumors have the less favourable outcome, this may be explained by the high percentage of invasive lobular carcinomas in this subgroup [10, 14].

## 5. Conclusion

 Although multifocal and diffuse invasive breast carcinomas exhibited a doubled frequency of LNM compared to that in unifocal tumors, no statistically significant differences could be demonstrated between these categories regarding histology grade, ER, PR, or HER2 status and regarding the proportion of tumors with basal-like phenotype. Multifocal and diffuse distribution of the invasive tumor component seems to be an independent negative morphologic prognostic parameter in breast cancer.

##  Conflict of Interests 

The authors declare no conflict of interests.

## Figures and Tables

**Table 1:** Immunophenotypic parameters, histology grade and node status by invasive tumor distribution (unifocal, multifocal, and diffuse) in 875 consecutive breast cancer cases, Falun 2005–2009.

Phenotype	Invasive tumor distribution				Significance level	
	Unifocal	Multifocal	Diffuse	Total	Unifocal versus multifocal	Unifocal versus diffuse
ER positive	83.1% (423/509)	81.8% (256/313)	97.7% (42/43)	83.4% (721/865)	*P* = .7852	*P* = .0178
PR positive	66.3% (331/499)	64.9% (203/313)	53.5% (23/43)	65.1% (557/855)	*P* = .8289	*P* = .1565
HER2 positive	9.6% (48/501)	15.2% (48/315)	4.8% (2/42)	11.4% (98/858)	*P* = .0419	*P* = .4334
Triple negative	11.2% (56/499)	10.5% (33/313)	2.4% (1/42)	10.5% (90/854)	*P* = .9083	*P* = .1144
Basal-like	12.5% (37/296)	11.3% (22/195)	3.7% (1/27)	11.6% (60/518)	*P* = .6020	*P* = .2896
Grade 3	22.6% (115/509)	25.2% (80/318)	7.0% (3/43)	22.8% (198/870)	*P* = .5664	*P* = .0245
Node positive	27.3% (140/513)	54.2% (173/319)	55.8% (24/43)	38.5% (337/875)	*P* < .0001	*P* = .0001
